# The Efficacy of 22 °C Static Subnormothermic Preservation with an Extracellular-Type Solution for 2 h Warm-Ischemic Porcine Kidneys

**DOI:** 10.3390/jcm14176156

**Published:** 2025-08-31

**Authors:** Akira Kondo, Masayoshi Okumi, Yuichi Ariyoshi, Mitsuhiro Sekijima, Akihiro Kawai, Takehiro Iwanaga, Kazuhiro Takeuchi, Kohei Miura, Shiori Miura, Akiyuki Iwamoto, Kenya Shimizu, Yurika Ichinari, Akira Shimizu, Mamoru Kusaka, Hisashi Sahara

**Affiliations:** 1 Division of Experimental Large Animal Research, Life Science and Laboratory Animal Research Unit, Center for Advanced Science Research and Promotion, Kagoshima University, Kagoshima 890-8520, Japan; meak07028@gmail.com (A.K.);; 2 Department of Urology, Kyoto Prefectural University of Medicine, Kyoto 602-8566, Japan; okumi@koto.kpu-m.ac.jp; 3 Department of Urology, Fujita Health University Okazaki Medical Center, Okazaki 444-0827, Japan; 4 Division of Digestive and General Surgery, Niigata University Graduate School of Medical and Dental Sciences, Niigata 951-8510, Japan; 5 Department of Research Ethics and Biorisk Management, Institute for Research Administration, Niigata University, Niigata 950-2181, Japan; 6 Department of Analytic Human Pathology, Nippon Medical School, Tokyo 113-8602, Japan

**Keywords:** static subnormothermic preservation, extracellular-type preservation solution, ischemia–reperfusion injury, normothermic machine perfusion, kidney transplantation, porcine

## Abstract

**Background**: Static cold storage is the standard method of kidney preservation following donation after circulatory death (DCD). A previous study on rodent models demonstrated the efficacy of storing DCD kidneys at 22 °C in an extracellular-type solution (ETK). We evaluated the efficacy of storing warm-ischemic kidneys at 22 °C in MHC-inbred miniature swine. **Methods**: After 2 h warm ischemia, the kidneys were preserved in ETK for one hour at either 4 °C or 22 °C and then subjected to ex vivo normothermic machine perfusion (NMP) for 2 h (*n* = 3 in each group). The same warm-ischemic kidneys, preserved in ETK (*n* = 3 in each group) or intracellular-type solution (UW; *n* = 2 in each group) at either 4 °C or 22 °C, were transplanted into MHC-matched recipients. **Results**: Compared with kidneys preserved at 4 °C, those preserved at 22 °C showed significantly better physiological and metabolic indices during ex vivo NMP. Furthermore, renal function was significantly higher in transplanted kidneys, and graft biopsies on postoperative day 4 showed more localized necrosis in the renal tubules when kidneys were stored at 22 °C. In contrast, recipient animals with kidneys stored in UW solution did not survive for more than 7 days. **Conclusions**: Two-hour warm-ischemic kidneys from miniature swine showed improved preservation at 22 °C than at 4 °C when an extracellular-type solution was used.

## 1. Introduction

The shortage of organ donors remains one of the greatest challenges faced by the transplant community worldwide. Donation after circulatory death (DCD) donors have the potential to satisfy the increasing need for transplantable organs [1,2]. However, DCD kidneys have higher risks of primary graft dysfunction or delayed graft function, with negative impacts on both short- and long-term graft survival following transplantation owing to the need for warm ischemia [3,4,5]. The increasing discard rates of DCD kidneys also underscore the need to improve the preservation methods for this pool of donated organs [6]. DCD kidneys are particularly vulnerable to endothelial and tubular injury and reperfusion-related dysfunction because they undergo a period of warm ischemia before preservation [1,2,3,4,5]. Therefore, the preservation interval, which is the main modifiable period between procurement and implantation, is a crucial aspect in improving early post-transplant performance [1,2,3,4,5,6].

Static cold storage (SCS) is the gold standard for organ preservation because it reduces metabolism and the oxygen demand and is simple and economically feasible [7]. However, during hypothermia, adenosine triphosphate (ATP) deficits accumulate rapidly, leading to reduced Na^+^/K^+^ ATPase activity, thereby contributing to increased intracellular sodium concentrations, associated with potentially lethal cell swelling [8]. In addition, cold preservation results in a poor oxygen supply, weak vasodilatation, and the delayed recovery of energy charges. Moreover, reperfusion following cold storage induces apoptosis and tissue fibrosis [9,10,11]. In particular, kidneys from DCD donors are more susceptible to cold preservation injuries [12].

Recently, various preservation methods have been examined to maintain the physiological and metabolic function of DCD organs. Normothermic preservation reduces vasoconstriction and prevents cell membrane stiffening in both endothelial cells and cellular components of the blood, which can prevent cellular edema in critical organs [13]. However, normothermic organ preservation has the disadvantage of increased metabolism and oxygen demands [14]. To reduce the metabolic rate while maintaining viability, several researchers have considered storage under room-temperature (subnormothermic) conditions. A previous study using a rodent model showed that static subnormothermic preservation at 23 °C with an extracellular-type solution yielded superior outcomes in rat kidneys after 1 h warm ischemia compared to conventional hypothermic preservation at 4 °C [15]. In addition, several reports have validated the effectiveness of subnormothermic machine perfusion using porcine kidneys with a warm ischemia time (WIT) of less than one hour [16,17,18,19].

In this translational study using large animals, we evaluated the efficacy of static subnormothermic storage (SSS) with an extracellular-type solution in porcine kidneys exposed to extended warm ischemia for up to 2 h. We focused on the following: (1) preoperative assessment via normothermic machine perfusion (NMP) to determine whether the kidneys could be used and (2) longitudinal assessment via a heterotopic kidney transplantation (KTx) model using major histocompatibility complex (MHC)-inbred CLAWN miniature swine.

## 2. Materials and Methods

### 2.1. Rationale and Clinical Relevance

SCS is the global standard for kidney preservation, and NMP is being introduced for DCD kidneys in some centers [7,12,14]. To reflect this practice, we used a DCD-relevant model consisting of 120 min of warm ischemia, included NMP for pre-implant functional assessment, and performed MHC-matched porcine KTx to minimize the alloimmune response with tacrolimus. This allowed for the evaluation of the effects of several preservation methods.

### 2.2. Animals

CLAWN miniature swine aged 4–6 months and weighing 14–16 kg were obtained from the Kagoshima Miniature Swine Research Center (Kagoshima, Japan). Animal care, housing, and surgery were performed in accordance with the guidelines and regulations of the Committee for Animal Research at Kagoshima University, Japan.

### 2.3. Experimental Design

Kidneys from 16 MHC-inbred CLAWN miniature swine were subjected to 2 h warm ischemia, followed by one hour of either SCS in a refrigerator at 4 °C (ETK4 group) or SSS in a water bath at 22 °C (ETK22 group) with an extracellular-type preservation solution (ETK: extracellular trehalose Kyoto; Otsuka Pharmaceutical, Tokushima, Japan). Warm ischemia was induced by placing the excised donor kidney in a water bath at 37 °C.

**Figure 1:**
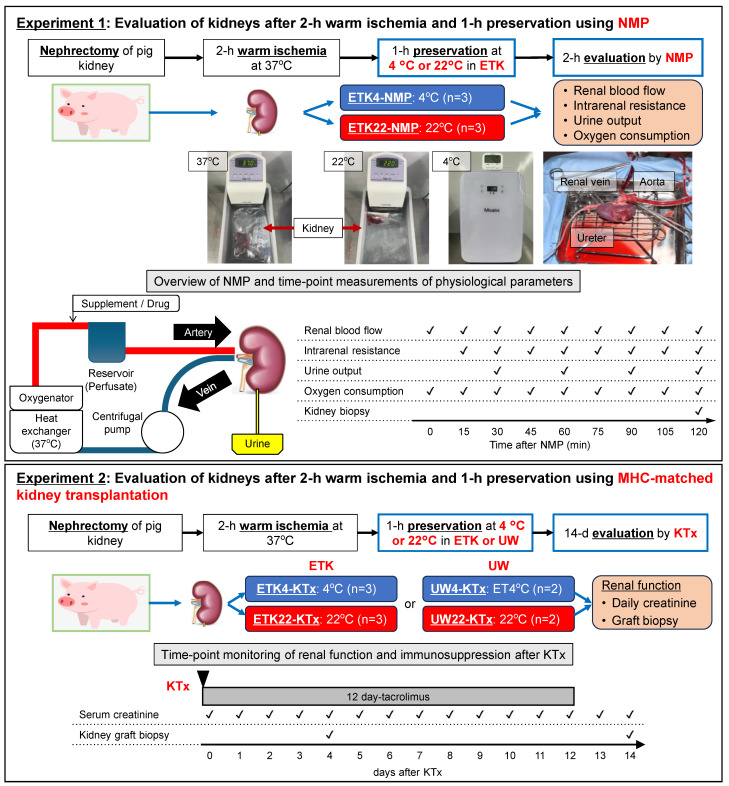
Experimental design for ex vivo normothermic machine perfusion (NMP) and MHC-matched kidney transplantation in a porcine model. Kidneys were subjected to 2 h of warm ischemia at 37 °C and then preserved for 1 h at either 4 °C or 22 °C using either an extracellular-type (ETK) (experiment 1 and 2) or intracellular-type UW (experiment 2) solution. To evaluate the kidneys after 2 h of warm and 1 h of cold preservation, NMP was performed in experiment 1 (upper panel) and MHC-matched kidney transplantation was performed in experiment 2 with tacrolimus for 12 days (lower panel). This technique-focused schematic summarizes the workflow and clarifies the experiment-specific evaluations. In experiment 1, NMP measurements included renal blood flow, intrarenal resistance, urine output, and oxygen consumption at 0–120 min, with renal biopsies performed at the end. In experiment 2, assessments included daily serum creatinine levels and protocol biopsies through day 14. Sample sizes (*n*) are indicated in the figure; no statistical analysis was applied in this workflow.

In experiment 1, preserved kidneys were subjected to ex vivo NMP with oxygenated perfusion solution for 2 h to assess preoperative kidney function (ETK4-NMP and ETK22-NMP; *n* = 3 in each group). In experiment 2, preserved kidneys were transplanted into MHC-matched recipients (ETK4-KTx and ETK22-KTx, *n* = 3 in each group). Post-transplant renal function, including serum creatinine (Cr) levels and kidney graft biopsies, was assessed over 14 days. In addition, 2 h warm-ischemic kidneys preserved in intracellular-type preservation solution (University of Wisconsin [UW] solution; Astellas Pharma Inc., Tokyo, Japan) at 4 °C and 22 °C for one hour were transplanted into MHC-matched recipients (UW4-KTx and UW22-KTx, *n* = 2 in each group) (Figure 1).

KTx and NMP procedures were performed by board-certified transplant surgeons (A.K., M.O., Y.A., M.S.), and histopathological assessment was conducted by renal pathology experts (K.T., A.S.) who were blinded to group allocation.

### 2.4. Normothermic Machine Perfusion (NMP)

**Figure 2:**
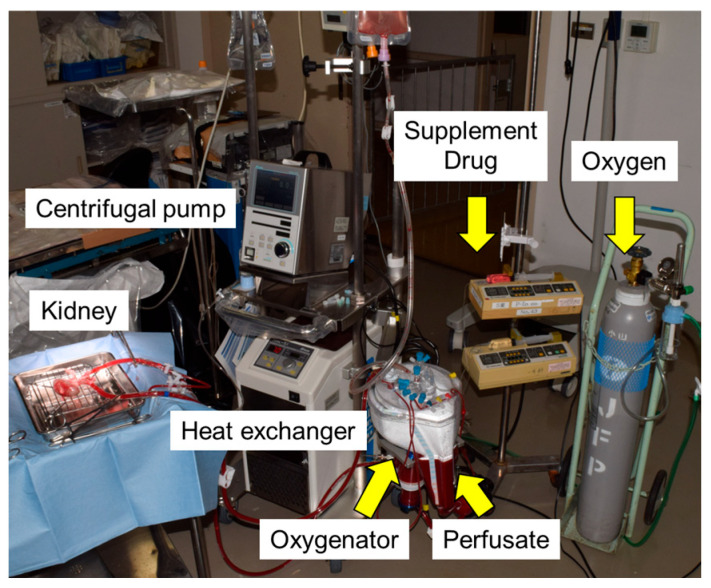
Setup for ex vivo normothermic machine perfusion of porcine kidneys.

After the renal artery, vein, and ureter were cannulated, preserved 2 h warm-ischemic kidneys were connected to the system and perfused with a normothermic perfusion solution for 2 h at a mean arterial pressure of 85 mmHg. The circuit was designed using a commercially available clinical-grade cardiopulmonary bypass system consisting of a centrifugal pump, heat exchanger, 5 L venous reservoir, and membrane oxygenator (CAPIOX, Terumo, Tokyo, Japan). The circuit hardware included a speed controller, flow transducer, pressure transducer, and temperature probe (Figure 2).

The perfusate consisted of 200 mL MHC-matched oxygenated red blood cells, 20 mL 20% mannitol, 8 mg dexamethasone, 30 mL 7% sodium bicarbonate, and 2000 units of heparin in 400 mL Ringer’s solution, as previously described by Hosgood et al. [20]. The perfusate was supplemented with 50 units of rapid-acting insulin, 5% glucose solution, 7% sodium bicarbonate, and 5 mL multivitamins to maintain the perfusate under physiological conditions. Prostacyclin was administered as a vasodilator during the first hour of reperfusion at 25 mL/h, followed by an infusion at 7 mL/h (Table 1).

For physiological and metabolic analyses, renal blood flow, mean arterial pressure, and blood gas parameters were monitored and recorded every 15 min, and urine output was recorded every 1 h (Figure 1). Intrarenal resistance and oxygen consumption were calculated as described by Hosgood et al. [20]. Renal biopsies were performed shortly after the termination of the 2 h NMP. The mRNA expression levels of IL-1β and IL-6 in the perfused kidneys were quantified using real-time PCR (RT-PCR), as described previously [21].

### 2.5. Surgeries (Catheter Placement, Kidney Transplantation, and Kidney Graft Biopsy)

Two semipermanent central venous catheters were placed in the external jugular veins of the recipient animals, and kidney transplantation was performed as described previously [22]. Protocol kidney graft biopsies were performed on postoperative days (POD) 4 and 14, respectively.

### 2.6. Immunosuppression

Tacrolimus (Astellas Pharma Inc., Tokyo, Japan) was continuously administered through an infusion pump for 12 days, starting on the day of kidney transplantation (day 0). The initial dose was 0.06 mg/kg/day. Whole-blood concentrations were measured at least every other day using a validated clinical assay, and the dose was titrated to maintain a target steady-state concentration of 15–25 ng/mL.

### 2.7. Assessment of Kidney Injury via Serum Analysis and Histological Examination

Serial serum Cr levels were assessed using venous blood samples obtained through a catheter from each experimental animal. Biopsy samples were prepared using 10% formalin fixation and paraffin embedding, followed by staining with hematoxylin and eosin (H&E) and proliferating cell nuclear antigen (PCNA) to assess tubular regeneration, as described previously [23]. All biopsy samples were assessed using standard light microscopy by a blinded pathologist.

### 2.8. Statistical Analysis

Statistical group comparisons were performed using either Student’s *t*-test, the Mann–Whitney U-test, or analysis of variance (ANOVA) using Prism 7 (GraphPad Software, Boston, MA, USA). The results are expressed as the standard error of the mean (SEM). Statistical significance was set at *p* less than 0.05.

## 3. Results

### 3.1. NMP Evaluation Revealed Superior Condition of 2 h Warm-Ischemic Kidneys Preserved at 22 °C Versus 4 °C (Experiment 1)

**Figure 3:**
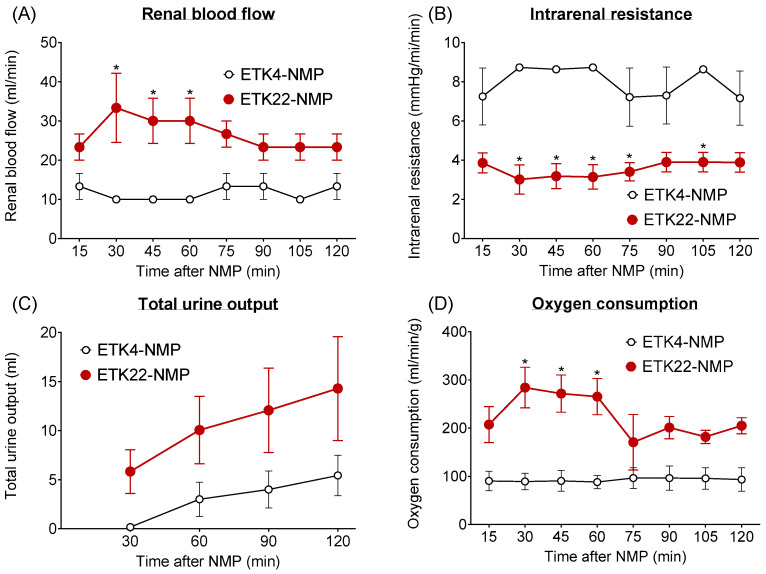
Physiological and metabolic parameters of kidneys subjected to 2 h warm ischemia and 1 h preservation during 120 min normothermic machine perfusion (NMP): (**A**) renal blood flow, (**B**) intrarenal resistance, (**C**) total urine output, (**D**) oxygen consumption. Kidneys were preserved for 1 h at either 22 °C (ETK22-NMP; red solid circles: *n* = 3) or 4 °C (ETK4-NMP; open circles: *n* = 3) in extracellular-type solution (ETK). The physiological and metabolic parameters of the ETK22-NMP group revealed superior condition compared to the kidneys in the ETK4-NMP group. The lines show the mean ± standard error of the mean. * *p* less than 0.05 between the ETK22-NMP and ETK4-NMP groups.

Using our established NMP method, all two-hour warm-ischemic kidneys preserved for one hour at either 4 °C or 22 °C with ETK solution were successfully perfused for two hours (ETK4-NMP and ETK22-NMP). Although all kidneys in both groups showed the same macroscopic appearance during NMP, the physiological and metabolic parameters of the kidneys in the ETK22-NMP group revealed their superior condition compared to those in the ETK4-NMP group (mean renal blood flow, 26.7 ± 4.7 vs. 10.0 ± 0.0 mL/min; mean intrarenal resistance, 3.5 ± 0.5 vs. 8.7 ± 0.1 mmHg/mL/min; total urine output, 14.3 ± 5.3 vs. 5.4 ± 3.6 mL; oxygen consumption, 223.5 ± 38.6 vs. 92.5 ± 15.6 mL/min/g; Figure 3).

**Figure 4:**
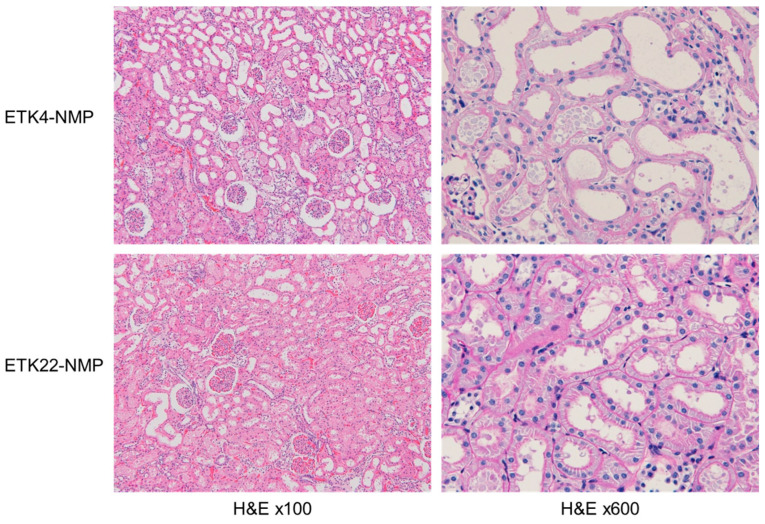
Histological findings for warm-ischemic kidneys after ex vivo normothermic machine perfusion. Diffuse tubular injury was observed in the 4 °C preservation group but not in the 22 °C group. Compared to the ETK22-NMP group, the ETK4-NMP group exhibited prominent histological features, such as the loss of brush borders, tubular cell flattening, and luminal dilation.

Histologically, diffuse tubular injury was observed in the 4 °C preservation group but not in the 22 °C preservation group. Compared with the ETK22-NMP group, prominent features were observed, including the loss of brush borders in the proximal tubules, cell flattening, and the dilation of the tubular lumen in the ETK4-NMP group (Figure 4).

**Figure 5:**
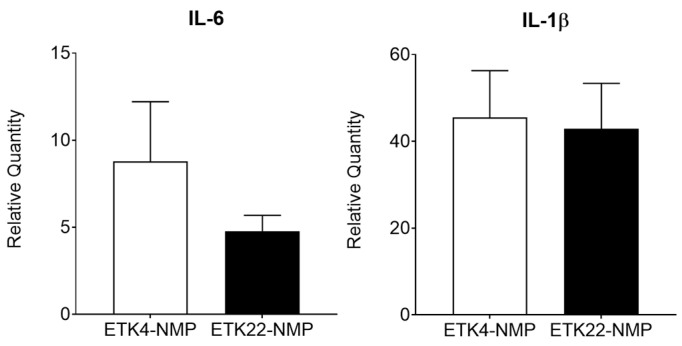
mRNA expression levels of inflammatory cytokines in kidneys subjected to 2 h warm ischemia, 1 h preservation, and 2 h normothermic machine perfusion (NMP) using RT-PCR. There were no statistical differences in the relative quantities of IL-6 and IL-1β at the end of the 2 h NMP in the 22 °C preservation group (ETK22-NMP; solid bar: *n* = 3) and 4 °C preservation group (ETK4-NMP; open bar: *n* = 3).

Next, we examined the mRNA expression levels of IL-6 and IL-1β in kidney specimens using RT-PCR. The relative quantities of IL-6 and IL-1β had increased at the end of the 2 h NMP in both groups, but there were no significant differences between the two groups (Figure 5).

### 3.2. Preservation of 2 h Warm-Ischemic Kidneys at 22 °C Resulted in Lower Serum Cr After MHC-Matched Kidney Transplantation (Experiment 2)

Based on the pretransplant NMP evaluation suggesting that kidneys preserved at 22 °C kidneys maintained a superior condition, we further evaluated the efficacy of 22 °C SSS on kidney grafts exposed to 2 h warm ischemia in a preclinical kidney transplantation model using MHC-inbred CLAWN miniature swine.

**Figure 6:**
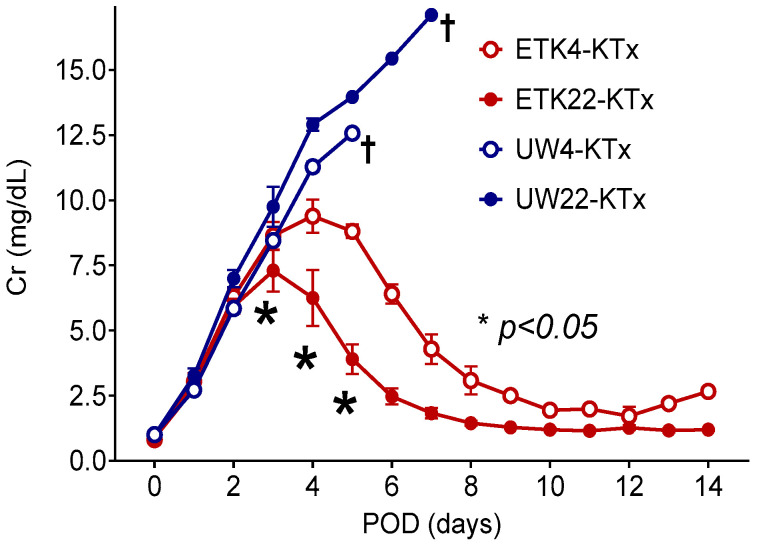
Serum creatinine levels following MHC-matched transplantation from kidneys subjected to 2 h warm ischemia and 1 h preservation with tacrolimus monotherapy. When preserved in an extracellular-type ETK solution, lower peak serum Cr levels and markedly earlier recovery of renal graft function were observed in the 22 °C preservation group (ETK22-KTx; red solid circle: *n* = 3) than in the 4 °C preservation group, regardless of whether kidneys were stored at 4 °C or 22 °C (ETK4-KTx; red open circle: *n* = 3). When preserved in an intracellular-type UW solution, regardless of whether kidneys were stored at 22 °C (UW22-KTx; blue solid circle: *n* = 2) or 4 °C (UW4-KTx; blue open circle: *n* = 2), none of the recipients survived for more than 1 week. * *p* less than 0.05 between the ETK22-KTx and ETK4-KTx groups. † Euthanasia before the endpoint.

Lower peak serum Cr levels and the markedly earlier recovery of renal graft function were observed on POD5 in the ETK22-KTx group compared to the ETK4-KTx group (3.9 ± 0.6 mg/dL vs. 8.9 ± 0.3 mg/dL, *p* = 0.0015) (Figure 6).

**Figure 7:**
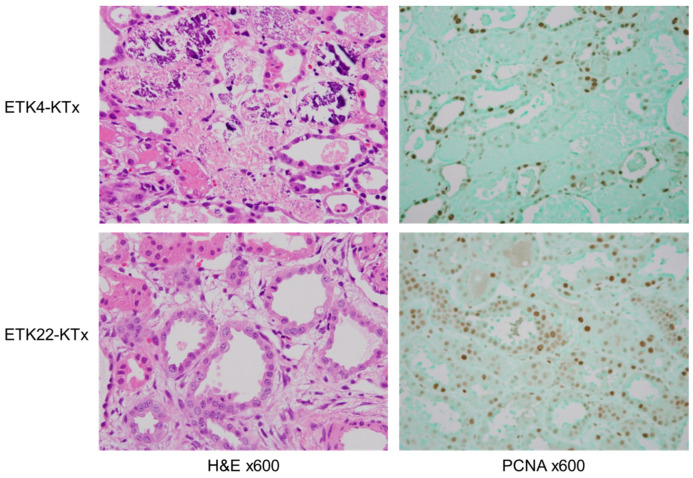
Histological and immunohistochemical evaluation of grafts on postoperative day 4. Biopsy specimens from the ETK4-KTx group showed widespread tubular necrosis, whereas from those the ETK22-KTx group exhibited limited necrosis. PCNA immunostaining revealed an increased number of PCNA-positive tubular cells in the ETK22-KTx group, indicating enhanced regeneration following acute tubular necrosis compared to that in the ETK4-KTx group.

Graft biopsy specimens obtained on POD4 from three animals in the ETK4-KTx group revealed the widespread necrosis of the renal tubules. However, the kidney grafts in the ETK22-KTx group showed limited necrosis of the renal tubules. We used immunohistochemical staining with PCNA to examine the regeneration and proliferation of tubular cells on POD4. Compared with the ETK4-KTx group, the number of PCNA-positive cells in the kidney grafts had increased in the ETK22-KTx group. This result indicates that SSS may encourage faster regeneration and recovery from acute tubular necrosis (ATN) compared to SCS (Figure 7).

### 3.3. Intracellular-Type Preservation Solution Could Not Preserve 2 h Warm-Ischemic Kidneys

To evaluate the efficacy of intracellular-type preservation solutions, we examined 2 h warm-ischemic kidneys preserved in UW solution at 4 °C and 22 °C for one hour, which were transplanted into MHC-matched recipients. However, none of the transplanted kidneys functioned, and none of the recipients survived for more than seven days in the UW22-KTx group or five days in the UW4-KTx group (Figure 6).

## 4. Discussion

In this study, using 2 h warm-ischemic kidneys, we demonstrated that SSS with an extracellular-type preservation solution was superior in maintaining the organs’ condition, as assessed via NMP for 2 h. Moreover, it significantly attenuated renal dysfunction and pathological damage induced by ischemia–reperfusion injury (IRI) in a large-animal model. Graft kidney biopsies performed 4 days after kidney transplantation revealed that ATN in SSS at 22 °C when using an extracellular-type preservation solution was weaker than that seen with traditional SCS. Moreover, the number of PCNA-positive cells, which indicates the rapid regeneration and proliferation of tubular cells, increased with SSS. These data suggest that SSS could reduce renal injury in 2 h warm-ischemic kidney grafts. Therefore, we conclude that it is convenient to use these short-term room-temperature conditions for transport purposes and storage prior to analysis in hospitals and diagnostic laboratories.

Several studies have demonstrated the beneficial effects of the subnormothermic preservation of warm-ischemic kidneys in large-animal models [16,17,18,19]. However, these studies have limitations, as they all used a perfusion system and the kidneys had a WIT of less than one hour. Furthermore, although there are reports describing KTx models, these consisted of autologous transplants that did not mimic clinical KTx. In this study, we demonstrate the effectiveness of preserving warm-ischemic kidneys at 22 °C using an allogeneic KTx model. To our knowledge, this is the first report to demonstrate that SSS can be applied to kidneys exposed to extended warm ischemia for as long as 2 h in a clinically relevant large-animal model to reduce renal IRI.

As we previously found that the effect of carbon monoxide reduced pulmonary IRI with a 90 min WIT in miniature swine [24], we also evaluated a 90 min WIT in our pilot study of the renal IRI model, but renal function only deteriorated slightly. Therefore, we concluded that a 90 min WIT could only produce a weak warm ischemia model and that a 2 h WIT would induce adequate renal IRI for further investigation. Furthermore, there is additional justification for the use of the IRI model with a 2 h WIT in a preclinical study. For instance, clinical studies have shown that DCD kidneys with less than a 2 h WIT, defined as the time between a systolic blood pressure of less than 50 mmHg and cardiac arrest, can be used for allogeneic kidney transplantation [1,25].

Therefore, because porcine kidneys exposed to extended warm ischemia for as long as 2 h were used in this study, we considered it important to investigate whether these kidneys could function before transplantation. Based on previous reports, we established the NMP method, as described above [20]. Using the NMP method, we found that SSS outperformed SCS. Moreover, the results obtained from the NMP model correlated with those from the KTx model. Thus, the NMP method can be used for preoperative evaluation in clinical settings.

Hypothermic preservation techniques were primarily developed for organs and tissue fragments intended for transplantation, where cryopreservation is complicated [26,27]. However, cooling and subsequent rewarming may contribute to apoptotic cell death and cause ultrastructural changes in renal tubular cells, similar to those observed in ATN, leading to renal failure [28,29]. Some researchers have demonstrated that cells and tissues can be stored more efficiently for shorter periods under subnormothermic conditions as compared to normothermic conditions due to lower metabolic rates [30,31]. In light of the above, SSS could allow for gradual rewarming and maintain viability while decreasing the metabolic rate, thus overcoming the disadvantages of other preservation methods. To explore the mechanism underlying the benefits of SSS, we assessed the differences in the production of inflammatory cytokines (IL-6 and IL-1β) in preserved kidneys in both groups, but we found no differences in the mRNA expression levels between them.

According to the Van’t Hoff principle, varying the temperature of preservation is expected to reduce tissue metabolism to 10–18% in hypothermic, 19–35% in midthermic, 36–85% in subnormothermic, and more than 86% in normothermic conditions [32]. In traditional cold preservation, low temperatures reduce the function of Na^+^/K^+^ ATPase, which causes an increase in intracellular Na +, resulting in high intracellular osmotic pressure; this in turn causes water influx and cell swelling. Renal Na^+^/K^+^ ATPase is particularly sensitive to ATP, and proximal tubular activity is proportional to the ATP concentration [33]. Thus, hypothermia inhibits the activation of Na^+^/K^+^ ATPase, causing severe injury to the proximal tubules of the kidney. Specific solutions containing macromolecular colloids, carbohydrates, antioxidants, and energy compounds are used for hypothermic storage to minimize the effects of IRI [34,35]. Therefore, solutions consisting of intracellular fluids with low Na concentrations, such as UW solution, are usually used for organ preservation. However, because this function is maintained during warm ischemia followed by warm preservation, superior results can be achieved by using preservation solutions with an extracellular fluid composition. Therefore, extracellular fluid is better adapted to preserve the marginal kidney than intracellular fluid in the normothermic state.

ETK solution, used for preservation in this study, has high sodium and low potassium levels, similar to the composition of extracellular fluid [36,37]. ETK contains trehalose and gluconate, which are two distinct components that exert cytoprotective effects and prevent cell swelling. A previous study reported that, compared with cold preservation using a traditional UW solution, a high-sodium version of UW decreased the glomerular filtration rate, urinary protein, interstitial fibrosis, and CD4+cell infiltration in a porcine kidney autotransplantation model [38]. The trehalose present in ETK solution prevents the denaturation of proteins, stabilizes protein structures and nucleic acids, and may inhibit the development of cellular edema [39,40,41]. In addition, Iwai et al. demonstrated that ETK solution was more beneficial than UW and intracellular trehalose Kyoto solutions in a rat kidney transplantation model using warm-ischemic kidneys preserved at 23 °C [15]. In this study, the ETK22-KTx group showed faster recovery from ATN than the ETK4-KTx group, and an additional experiment using UW solution at either 4 °C or 22 °C showed no recovery from ATN in 2 h warm-ischemic kidneys. Our data strongly support the previously mentioned findings, and SSS with a trehalose-containing solution is beneficial in maintaining the quality of marginal kidneys.

One of the major obstacles in clinical transplantation is the shortage of donor organs. Organs from extended-criteria donors or marginally ischemic organs from DCD donors are required to bridge the gap between the supply and demand of organs for transplantation. However, these organs are susceptible to severe IRI, which can lead to a high incidence of delayed graft function or primary nonfunction during the early perioperative period. Subnormothermic machine perfusion at temperatures of 20–25 °C could allow the elimination of hypothermia-induced injury while utilizing established techniques and perfusate solutions, without the need for oxygen carriers (e.g., erythrocytes). Nevertheless, organ perfusion is limited by the availability and cost of perfusion systems, the need for perfusate solutions, and the risk of infection. Additionally, perfusion machine failure can result in graft loss. In the present study, we demonstrate that SSS is sufficient to restore kidney function following kidney transplantation. However, a limitation of this study is that the preservation time was only one hour, whereas, in previous studies, the perfusion time was four hours or more.

Several possible future applications in clinical practice can be considered in appropriate settings. When warm ischemia is prolonged and transplantation occurs in the same center, or when rapid NMP is planned soon after procurement, the routine step of cooling the graft to 4 °C may be counterproductive. In our setting, brief static storage at 22 °C in ETK preserved the graft conditions before NMP and was associated with earlier post-transplant recovery, suggesting that the preservation temperature should be selected according to the situation, rather than being applied uniformly.

This study has some important limitations. Firstly, it was a single-center porcine DCD study with a limited number of animals per group (particularly in the UW arms) and a short static storage interval (1 h). Secondly, warm ischemia was applied for a longer period than is typically anticipated in clinical practice; therefore, extrapolation to situations with shorter time periods requires caution. Thirdly, because ETK is not commonly used for clinical kidney transplantation, the observed differences from UW require mechanistic clarification to disentangle the respective contributions of the solution composition and temperature to tubular and endothelial protection. Fourthly, we did not evaluate other subnormothermic temperatures or compare perfusion strategies in adequately powered cohorts. Future studies should address these points and deftermine whether similar benefits are observed with shorter periods of warm ischemia and alternative subnormothermic conditions.

## 5. Conclusions

In conclusion, in a porcine DCD model with 2 h of warm ischemia, SSS at 22 °C using an extracellular-type solution preserved graft quality to a greater degree than conventional 4 °C SCS, yielding earlier and more sustained functional recovery, reduced tubular injury, and greater PCNA-positive regeneration. Preservation with the UW solution at either temperature did not maintain graft function, and the ex vivo NMP results were consistent with the in vivo outcomes. These findings may inform temperature choices in settings with short procurement-to-NMP intervals or same-center transplantation. Limitations include the short preservation window, small group sizes, and the WIT, which was longer than that typically observed in clinical settings. Future studies should address these points and determine whether similar benefits are observed with longer preservation intervals, shorter warm ischemia periods, and alternative subnormothermic conditions.

## Figures and Tables

**Table 1:** Composition of perfusate used for normothermic machine perfusion.

Perfusate Component	Quantity
Red blood cells	200 mL
Ringer’s solution	400 mL
20% D-Mannitol	15 mL
Dexamethasone	8 mg
7% Sodium bicarbonate	30 mL
Heparin sodium	2000 IU
**Circuit supplement or drug**	**Dosing (initial dose or infusion rate)**
Prostacyclin	25 mL/h
5% Glucose	15 mL/h
7% Sodium bicarbonate	15 mL/h
Insulin	50 units
Multivitamins	5 mL

## Data Availability

The original contributions presented in this study are included in this article. Further inquiries should be directed to the corresponding author.

## Abbreviations

The following abbreviations are used in this manuscript:

ATN	Acute tubular necrosis
ATP	Adenosine triphosphate
DCD	Donation after circulatory death
ETK	Extracellular trehalose Kyoto
IRI	Ischemia–reperfusion injury
KTx	Kidney transplantation
MHC	Major histocompatibility complex
NMP	Normothermic machine perfusion
PCNA	Proliferating cell nuclear antigen
POD	Postoperative day
RT-PCR	Real-time PCR
SCS	Static cold storage
SSS	Static subnormothermic storage
PGD	Primary graft dysfunction
UW	University of Wisconsin
WIT	Warm ischemia time
